# The colonization of pyrethroid resistant strain from wild *Anopheles sinensis*, the major Asian malaria vector

**DOI:** 10.1186/s13071-014-0582-7

**Published:** 2014-12-11

**Authors:** Guoding Zhu, Huayun Zhou, Julin Li, Jianxia Tang, Liang Bai, Weiming Wang, Yaping Gu, Yaobao Liu, Feng Lu, Yuanyuan Cao, Chao Zhang, Sui Xu, Jun Cao, Qi Gao

**Affiliations:** Key Laboratory of Parasitic Disease Control and Prevention (Ministry of Health), and Jiangsu Provincial Key Laboratory of Parasite Molecular Biology, Jiangsu Institute of Parasitic Diseases, Wuxi, Jiangsu Province People’s Republic of China; Department of Parasitology, Medical College of Soochow University, Suzhou, 215123 People’s Republic of China; Public Health Research Center, Jiangnan University, Wuxi, People’s Republic of China

**Keywords:** *Anopheles sinensis*, Pyrethroid, Insecticide resistance

## Abstract

**Background:**

*Anopheles sinensis* is one of the most important malaria vectors in Asian countries. The rapid spread of insecticide resistance has become a major obstacle for insecticide-based strategies for vector control. Therefore, it is necessary to prepare an insecticide-resistant strain of *An. sinensis* to further understand the insecticide resistance mechanisms in this species to facilitate genetic approaches to targeting the insecticide-resistant population of this important malaria vector.

**Methods:**

*An. sinensis* mosquitoes were collected from regions where pyrethroid resistance had been reported. The mosquitoes were subjected to continuous pyrethroid selection after species confirmation, and the forced copulation method was used to increase the mating rate. In addition, the knockdown-resistance (*kdr*) mutation frequencies of each generation of *An. sinensis* were measured; and the metabolic enzyme activities of cytochrome P450 monoxygenases (P450s) and glutathione S-transferases (GSTs) were detected.

**Results:**

The identification of field-captured *An. sinensis* was confirmed by both morphological and molecular methods. The population of *An. sinensis* exhibited stable resistance to pyrethroid after continuous generations of pyrethroid selection in the laboratory with high *kdr* mutation frequencies; and elevated levels of both P450s and GSTs were significantly found in field selected populations comparing with the laboratory susceptible strain. So far, the colonised strain has reached its eleventh generation and culturing well in the laboratory.

**Conclusions:**

We colonised a pyrethroid-resistant population of *An. sinensis* in the laboratory, which provides a fundamental model for genetic studies of this important malaria vector.

## Background

*Anopheles sinensis* (Diptera, Culicidae) is one of the major malaria vector mosquitoes in China and other Asian countries [1-4]. This species is an important member of the *An. hyrcanus* group (Diptera, Culicidae) with similar morphology [5]. Increased attention has been paid to this species due to its wide distribution, high abundance and modest susceptibility to malaria parasites reported in previous studies [6,7].

Vector control is defined by the World Health Organization (WHO) as one of the four basic and most effective measures to prevent malaria transmission and remains a component of malaria control strategies, including in the elimination stage [8]. The intervention based on indoor residual spraying (IRS) is the most widely adopted method in almost all regions in China at risk of malaria transmission. Pyrethroids are often used for IRS due to their relative long persistence and low toxicity in comparison with the other three major classes of available insecticides [9]. However, the substantial increase in pyrethroid-based malaria vector control in the past decade has resulted in the rapid spread of resistance among malaria vectors. Furthermore, the widespread use of these compounds for agricultural purposes has further accelerated the development of insecticide resistance [10]. High-level resistance to pyrethroids has been reported in *An. sinensis*, especially in central China, in recent years, which has placed current national efforts of malaria elimination at risk [11]. As a result, improvements to the surveillance of and response to insecticide resistance in *An. sinensis* in China are urgently needed and must be based on a greater understanding of the molecular mechanisms underlying this resistance.

Genetic approaches, such as genetic linkage studies and QTL mapping [12-14], are useful tools for understanding insecticide resistance mechanisms and developing a method for monitoring insecticide resistance in both the susceptible and resistant strains. Our laboratory has successfully colonised the susceptible *An. sinensis* strain; more than five hundred generations of this strain have never been exposed to any insecticide, dating back to the 1980s. To prepare an insecticide-resistant strain in the laboratory, in 2013, we collected wild *An. sinensis* from areas with reports of high resistance levels in recent years [15] for use in breeding field-derived, insecticide-resistant *An. sinensis* in the laboratory. The knockdown-resistant (*kdr*) mutation frequencies among continuous generations, and the metabolic enzyme activities were also investigated.

## Methods

### Mosquito colonisation

**Figure 1:**
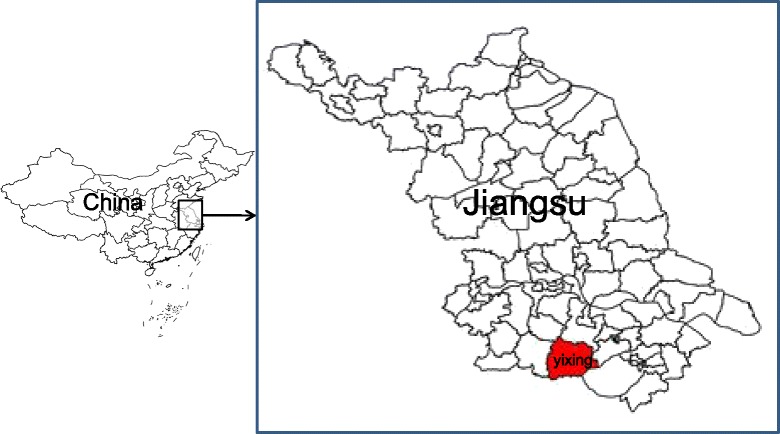
**Study site for field mosquito collection in 2013.**

From June to July in 2013, living engorged female anopheline mosquitoes (F0) were captured gently with a mouth aspirator in pigsties near a rice field in Yixing County (119°38′ E, 31°16′ N), Jiangsu Province, China, where *Plasmodium vivax* has been the only prevalent species of malaria in recent years (Figure 1). Subsequently, all of the mosquitoes were transported to the insectary of the Key Laboratory on Technology for Parasitic Disease Control and Prevention, Ministry of Health, Jiangsu Institute of Parasitic Diseases (JIPD) in Wuxi, Jiangsu Province, China. The insectary is maintained at 26 ± 1°C, 70–80% relative humidity with a 12 h day/night lighting regime, and the mosquitoes were provided with 10% (w/v) glucose in water. Distilled water-saturated filter paper was placed in the mosquitoes’ cage, and the females were allowed to oviposit. Subsequently, the eggs were morphologically identified to avoid potential contamination with the other similar anopheline mosquitoes of the *An. hyrcanus* group, such as *An. anthropophagus* (Diptera, Culicidae), which closely resembles *An. sinensis* in the adult stage but differs morphologically in the egg stage. All families identified as *An. sinensis* were pooled, and larvae were reared to adults (first generation, F1). The larvae were fed finely ground tropical fish food (TetraMin, Germany). Newly emerged female and male adults were placed in separate containers to ensure that mating did not take place prior to exposure to insecticides. Randomly selected F1 adults were identified using the *rDNA ITS*_*2*_-based method to further confirm the species’ identity [16].

### Pyrethroid-resistance selection

All the 3- to 5-day-old newly emerged female and male adults, approximately twenty-five mosquitoes per exposure tube, from each generation were exposed to 0.05% deltamethrin-treated papers for 1 h according to the standard WHO bioassay procedure (WHO, 1998). WHO (2013) criteria were used to classify the resistance status of the tested mosquito population after 24 h recovery from the insecticide [17]. All of the male and female survivors 24 h post-exposure were placed in separate cages, and the females were left for blood-feeding followed by forced mating with the male survivors of similar age. After two days, water was added to the filter paper, and female mosquitoes were allowed to oviposit; second bloodmeals were offered to allow the females to oviposit again [18]. Eggs from each generation were reared to adults and selected for resistance to deltamethrin in the same manner until a stable resistance level was reached, after which insecticide selection with a generation interval was used.

### Forced mating

To overcome the obstacle of the low rate of mating resulting from the shift to the new environment among the field-collected mosquitoes in the laboratory, the forced mating method based on MR4 was used to increase the mating rate [19]. Briefly, the female mosquitoes living after 24 h recovery from the exposure to deltamethrin were blood-fed with an anaesthetised mouse. The engorged female mosquitoes were anaesthetised by ethyl ether 4–5 h post-blood-feed; the well-fixed males were decapitated to overcome the potential innate inhibition of their copulatory muscles. The males were placed at an angle venter-to-venter with the female until the males clasped the females and mated successfully.

### gDNA extraction and *kdr* genotype identification

The total genomic DNA of individual mosquitoes was extracted using QIAGEN DNeasy 96 Kits according to the manufacturer’s instructions. Briefly, one or two mosquito legs were added to a mixture of 100 μL of Tissue Lysis Solution and 10 μL of Proteinase K and were incubated at 55°C for 10 min and 95°C for 10 min. Next, 100 μL of Neutralization Solution T was added to the mixture, which was then centrifuged at 17,900 × g for 3 min. The supernatant was transferred to a new tube and stored at −30°C for species identification and kdr detection. The DNA extracts of randomly selected F1 mosquitoes were amplified for species determination using an *rDNA-ITS*_*2*_ based method. PCR products were visualised under ultraviolet light after electrophoresis using 2% agarose gel stained with ethidium bromide. DNA extracts of female mosquitoes from F0 to F11 were genotyped individually using a recently developed TaqMan-MGB probe assay for *kdr* mutation detection at codon position 1014 of the para-type sodium channel [20,21] and run on a LightCycler 480 qPCR thermal cycler (Roche). The PCR conditions were an initial denaturation step of 10 min at 95°C followed by 40 cycles of 95°C for 10 s and 65°C for 15 s.

### Metabolic enzyme activity detection

The 3–4 days post emergence female mosquitoes alive 24 h after 60 min exposure to insecticide from F11, and the mosquitoes knocked down from colonized laboratory susceptible populations during the 60 min exposure were selected, to ensure only the fresh mosquitoes were immediately tested for metabolic enzyme activities. The selected individual mosquitoes were homogenized with phosphate KPO_4_ buffer (0.25 M, pH 7.2), and diluted by adding phosphate buffer, and then the supernatant was used to test the enzyme activities of cytochrome P450 monoxygenases (P450s) and glutathione S-transferases (GSTs) based on our previously published protocol [22]. Mean absorbance values for each tested mosquito and enzyme were converted into enzyme activity and standardized based on the total protein amount. All measurements were performed in duplicate.

### Statistical analysis

The Chi-square test was used to compare the mortalities between the sexes and the generations. The t-test was used to determine whether monooxygenases and GST activity varied between the laboratory strain and the field *An.sinensis* mosquitoes.

### Ethical approval of animal use

The animal experiments were approved by the Jiangsu Institutional Animal Care and Use Committee (IACUC), according to the guideline of administration of lab animals issued by the Ministry of Science and Technology (Beijing, China). All animal procedures were approved by the Institutional Review Board (IRB00004221) of Jiangsu Institute of Parasitic Diseases (Wuxi, China).

## Results

### Mosquito species confirmation and colonisation

**Figure 2:**
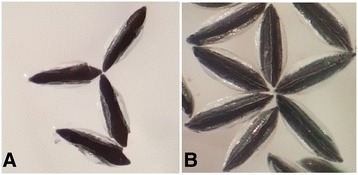
**Morphological characteristics of the eggs of** ***An. sinensis*** **and** ***An. anthropophagus*** **, observed under a normal dissection microscope. (A)**. The wide float in *An. sinensis*. **(B)**. The narrow float in *An. anthropophagus*.

From June to July, we collected more than 2000 engorged anopheline mosquitoes. All of the batches of eggs laid by the parents (F0) were morphologically confirmed as *An. sinensis*, which exhibit a relatively wider float than the considerably narrower one in *An. anthropophagus* (Figure 2). *Anopheles.anthropophagus* has historically been distributed from limited areas in Jiangsu Province. The randomly selected F1 adults were further confirmed as *An. sinensis* using the *rDNA-ITS*_*2*_ method [16].

### Pyrethroid resistance selection

Male and female F1 *An. sinensis* mosquitoes were separately tested with a standard WHO bioassay, and the resistance status of both sexes was classified as “Resistant”. As shown in Table 1, the mortality decreased significantly in males (χ2 = 51.79, *ν* = 2, *p* < 0.01) and females (χ2 = 28.07, *ν* = 2, *p* < 0.01) after the deltamethrin selection process from F1 to F3. After F4, both the male and female populations exhibited zero mortality. The females were more resistant to the deltamethrin treatment than the males in the F1 (χ2 = 16.98, *ν* = 1, *p* < 0.01) and F2 (χ2 = 22.52, *ν* = 1, *p* < 0.01) generations; however, no significant difference was observed in the F3 generation (χ2 = 0.55, *ν* = 1, *p >* 0.05), and both sexes were completely resistant to deltamethrin after F4. The laboratory *An. sinensis* mosquitoes, by contrast, were all killed after exposure to the same dose of deltamethrin (Table 1).

**Table 1:** **Mortality upon exposure to deltamethrin in** ***An. sinensis*** **populations**

**Population**		**Sample size (n)**	Mortality ± SE (%)*
Field-F1	Male	570	14.08 ± 0.48
	Female	470	6.21 ± 0.21
Field-F2	Male	469	11.86 ± 2.21
	Female	495	3.66 ± 0.98
Field-F3	Male	402	0.84 ± 0.27
	Female	510	0.27 ± 0.12
Field-F4	Male	350	0.00
	Female	405	0.00
Field-F5	Male	251	0.00
	Female	283	0.00
Field-F7	Male	262	0.00
	Female	292	0.00
Field-F9	Male	151	0.00
	Female	162	0.00
Field-F11	Male	135	0.00
	Female	148	0.00
Laboratory**	Male	100	100
	Female	100	100
Control***		40	0

*Mortality refers to the percentage of mosquitoes that died 24 h after recovery from a 60 min exposure to the insecticides. Resistance was defined as mortality <90%, probable resistance was defined as mortality 90–98%, and susceptibility was defined as mortality >98%. **The laboratory mosquitoes included the susceptible colony, which has been cultured in the insectaria for more than 500 generations and has never been exposed to any insecticide. ***Forty mosquitoes of laboratory strain were exposed to filter paper without insecticide for 60 min, and mortality was recorded after the 24 h recovery period.

To date, this colonised strain has reached its eleventh generation. *An. sinensis* mosquitoes from F1 to F5 were tested with the standard WHO bioassay, followed by the same treatment in F7, F9 and F11, when exhibited stable resistant to the deltamethrin (Table 1).

### *kdr* genotype

Two types of *kdr* mutations at codon position 1014 of the para-type sodium channel gene among the randomly selected female field *An. sinensis* were detected (Table 2): one mutation from TTG to TTT that causes a leucine to phenylalanine substitution (L1014F) and one mutation from TTG to TGT that causes a leucine to cysteine substitution (L1014C). The original population collected from the field (F0) had a high mutation frequency (98.73%), with a dominant *kdr* mutation of L1014F. Similarly, all of the subsequent generations from the field F1 to F4, F5 and F7 after the deltamethrin selection exhibited a high mutation frequency, and the individuals in F2, F3, F4, F5 and F7 exhibited a 100% mutation frequency. The dominant *kdr* mutation was L1014F in the F3 generation. However, no *kdr* mutation was detected in the susceptible laboratory strain (Table 2).

**Table 2:** **Distribution of** ***kdr*** **allele frequencies in** ***An. sinensis*** **populations**

**Population**	**Wild type**	**Mutation**					**Mutation frequency (%)**
	**TTG/TTG**	**TTT/TTT**	**TGT/TGT**	**TTT/TGT**	**TTG/TGT**	**TTG/TTT**	
Field-F0	0	102	14	38	0	4	98.73
Field-F1	0	88	16	48	4	6	96.91
Field-F2	0	58	60	24	0	0	100
Field-F3	0	102	0	0	0	0	100
Field-F4	0	70	0	0	0	0	100
Field-F5	0	81	0	0	0	0	100
Field-F7	0	68	0	0	0	0	100
Field-F9	0	72	0	0	0	0	100
Field-F11	0	78	0	0	0	0	100
Laboratory	63	0	0	0	0	0	0

### Metabolic enzyme activities

**Figure 3:**
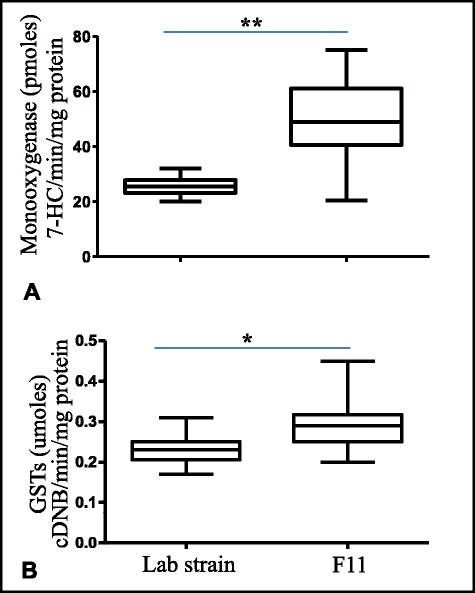
**Boxplots of metabolic enzyme activities in** ***An. sinensis*** **from laboratory susceptible strain and field-selected, F11.** The median activity is shown by horizontal bar; the upper and lower quartiles is shown by the box, and the full range of the data set is shown by the vertical lines. *,*p* < 0.05; **,*p* < 0.01. **A**: P450 monooxygenases; **B**: GSTs.

100 and 60 randomly selected female *An.sinensis* from laboratory susceptible strain and F11, respectively, were tested for metabolic enzyme activities. The median GST activity of the lab strain was 0.231 μmol cDNB/min/mg protein (ranging from 0.17 to 0.31), and the median P450 activity was 25.5 pmol 7-HC/min/mg protein (ranging from 19.9 to 31.9). Comparing with the lab susceptible strain, significantly elevated levels of both the GSTs and P450s were found in field-selected mosquitoes, F11 (Figure 3).

## Discussion

The establishment of resistant strains is essential for further understanding the mechanism of the development of insecticide resistance among insects, including anopheles mosquitoes; the colonised resistant strains could also be used in testing new malaria interventions/tools that might counter physiological resistance among these vector species. As we already possessed the deltamethrin-susceptible strain of *An. sinensis* in the laboratory, we previously attempted to induce and then select the deltamethrin-resistant strain from this colony. However, the median lethal concentrations (LC_50_s) after generations of exposure to deltamethrin in 3rd and 4th instar larvae mosquitoes decreased to the original level after only a gradual 1-3-fold increase over several generations. This phenomenon differed greatly from the insecticide selection in other laboratory colonised species, *Culex pipiens pallens*, in which the LC50 to deltamethrin rapidly increased to approximately five-hundred times the original value after ten continuous generations of exposure [23]. The distinctly different outcomes of deltamethrin selection between *An. sinensis* and *Culex pipiens pallens* may be due to the species variation, a possibility that requires further investigation. Compared with the laboratory colony, wild mosquito populations are under continuous selection pressure from insecticide-associated activities related to public health and agriculture [24,25]. As a result, the susceptible individuals in these populations decrease while resistant individuals increase [26]. The annual report from vector monitoring and surveillance systems demonstrated that a high level of insecticide resistance in wild populations has developed. Therefore in the present study, we collected wild *An. sinensis* and applied deltamethrin as a selection agent instead of applying the selection process to the laboratory colony.

To our knowledge, this is the first description of selecting for deltamethrin resistance and maintaining an *An. sinensis* colony by forced mating. As has been well established, the greatest obstacle to natural mosquito colonisation is the low mating rate due to the switch from the natural environment to the laboratory [27]; the mosquitoes prefer to swarm in open areas rather than mate in narrow cages, especially in the first several generations. Indeed, we failed to colonise this species in 2012 because our specimens failed to produce sufficient progeny after several generations of selection by insecticides. To overcome this difficulty, the forced mating method was used in the present study. The forced mating method is still being used to maintain the colony due to the amount of the population. It is important to note that healthy males and females are critical for successful mating. Other practices can also be helpful, including allowing the engorged female mosquitoes at least 3–5 h of digestion time prior to mating instead of mating immediately after blood feeding and using males of a similar age or younger than the females. In the addition, although a bloodmeal feeding to females prior to artificial mating may not be necessary, previous research has indicated that the engorged abdomen of the female facilitates forced copulation [28].

Given that the wild mosquitoes already exhibited a high level of resistance to pyrethroids, indicating a very low proportion of susceptible individuals living in the natural populations, we were not surprised to find that the mortality of the progeny of the wild *An. sinensis* reached zero after only three continuous generations of selection by removing the susceptible individuals in the population. The proportions of both male and female progeny originating from the natural populations tended to increase with deltamethrin resistance after selection by the insecticide. The female cohorts in the first two (F1 and F2) selected generations exhibited markedly higher levels of resistance than their male counterparts, which demonstrated a potential distortion based on the sex-linked factor associated with resistance. However, it should be noted that selection for resistance after the third generation (F3) revealed high levels of survival among females as well as males, which was also supported the results of a previous study on insecticide selection in field-collected *An. funestus*, which is an important malaria vector in Africa [29].

The mutation at position 1014 causing a change from leucine to either phenylalanine (L1014F) or serine (L1014S), conferring knockdown-resistance (*kdr*), was the most common among the identified point mutations in the para-type sodium channel gene in anopheline mosquitoes associated with pyrethroid resistance [22,30]. In the present study, the natural population (F0) of *An. sinensis* included a large sub-population with the *kdr* mutation, among which L1014F and L1014C were identified. However, we did not detect any L1014S mutation, unlike previous reports on *An. sinensis* populations from other regions [31]. Furthermore, the *kdr* allele (TTG to TTC) leading to L1014F, which was previously observed in central China, was not detected [32], which indicates that the *kdr* mutation might be largely determined by sampling factors and might differ markedly in different areas. The dominant *kdr* mutation was the L1014F mutation during the process of selection, with an exception observed only in the F2 generation, consistent with previous results in *Culex pipiens pallens* mosquitoes subjected to deltamethrin selection [33]. We did not detect any *kdr* mutation in the laboratory mosquitoes susceptible to deltamethrin compared with the resistant-phenotype field mosquitoes with high *kdr* mutation status, suggesting that the *kdr* mutation might play an important role in the formulation of insecticide resistance in this species. However, the continuous increasing level of resistance from F2 to the subsequent generations despite the 100% *kdr* mutation frequencies achieved as early as F2 suggests that other issues are also involved in insecticide resistance in addition to the *kdr*. In the present study, we found both the levels of enzyme activity in P450s and GSTs were elevated in field insecticide-selected populations, with comparison to laboratory susceptible stain, in which more significantly elevation was found in P450s, supported by the recent studies in the field *An. sinensis* in central China [34,35], suggesting the metabolic detoxification especially the modification of P450s activity also play important role in the deltamethrin resistance in *An. sinensis*, besides the kdr mutation [36,37]. The colonised population of *An. sinensis* provides a valuable tool for further genetic approaches, e.g. to screen for the major gene loci among P450s conferring the deltamethrin resistance via QTL mapping using this colonised population and the susceptible strain, and to develop the subsequent monitoring method for insecticide resistance detection in this important species.

## Conclusions

The established colonised population of *An. sinensis* from the field exhibited a high resistance level to deltamethrin with the dominant *kdr* mutation allele L1014F and elevated detoxification enzyme activities. This population serves as a valuable research tool for future genetic studies in this important malaria vector to further elucidate the mechanism of insecticide resistance, and also for testing new malaria interventions/tools that might counter physiological resistance in this species.
